# Early warning signals of malaria resurgence in Kericho, Kenya

**DOI:** 10.1098/rsbl.2019.0713

**Published:** 2020-03-18

**Authors:** Mallory J. Harris, Simon I. Hay, John M. Drake

**Affiliations:** 1 Odum School of Ecology, University of Georgia, Athens, GA 30602, USA; 2 Center for the Ecology of Infectious Diseases, University of Georgia, Athens, GA 30602, USA; 3 Biology Department, Stanford University, 371 Serra Mall, Stanford, CA, USA; 4 Institute for Health Metrics and Evaluation, University of Washington, Seattle, WA 98121, USA; 5 Department of Health Metrics Sciences, School of Medicine, University of Washington, Seattle, WA 98121, USA

**Keywords:** malaria, forecasting, infectious disease, resurgence, statistics

## Abstract

Campaigns to eliminate infectious diseases could be greatly aided by methods for providing early warning signals of resurgence. Theory predicts that as a disease transmission system undergoes a transition from stability at the disease-free equilibrium to sustained transmission, it will exhibit characteristic behaviours known as critical slowing down, referring to the speed at which fluctuations in the number of cases are dampened, for instance the extinction of a local transmission chain after infection from an imported case. These phenomena include increases in several summary statistics, including lag-1 autocorrelation, variance and the first difference of variance. Here, we report the first empirical test of this prediction during the resurgence of malaria in Kericho, Kenya. For 10 summary statistics, we measured the approach to criticality in a rolling window to quantify the size of effect and directions. Nine of the statistics increased as predicted and variance, the first difference of variance, autocovariance, lag-1 autocorrelation and decay time returned early warning signals of critical slowing down based on permutation tests. These results show that time series of disease incidence collected through ordinary surveillance activities may exhibit characteristic signatures prior to an outbreak, a phenomenon that may be quite general among infectious disease systems.

## 1. Introduction

Despite modern advances in disease control, the World Health Organization reports that nearly one-third of deaths in developing countries are due to infectious diseases [1]. So far, only one human global disease eradication campaign has been successful (smallpox). Especially in low-resource settings, premature abandonment of disease elimination programmes as other health priorities compete for limited resources may result in reestablishment, even when the disease is on the brink of elimination. For instance, in Sri Lanka, malaria incidence climbed from 17 to 440 644 cases between 1963 and 1968 [2,3]. Indeed, malaria resurgence occurred repeatedly throughout the last century. A systematic review identified 75 resurgence events worldwide between 1930 and 2000, attributed largely to the weakening of malaria control programmes, increasing the potential for transmission and evolution of drug and insecticide resistance [2]. Exactly those conditions are found for other diseases now on the cusp of global eradication (e.g. polio [4], Dracunculiasis [5] and human Africa trypanosomiasis [6]). It follows that effective disease elimination might be guided by techniques for differentiating between efforts that will lead to stable disease elimination and scenarios where cases are diminished without a corresponding shift in the system's stability. Few methods are available that are appropriate to the relevant problems of limited data and non-stationary disease dynamics at these transition points.

Forecasting methods that depend directly on raw case incidence have been proposed for infectious diseases, but techniques such as the moving epidemic method, which establishes thresholds for identifying the start of an epidemic, rely on regular, seasonal trends and are therefore not applicable for predicting the resurgence of vector-borne diseases [7]. Recent theory suggests that critical transitions in infectious disease systems may be detected using non-parametric statistical methods [8,9]. Resurgence is a type of critical transition, wherein the stable state of the disease system shifts from the disease-free equilibrium (where cases are due to imported infections and subcritical stuttering chains of local transmission) to self-sustained transmission. In systems where the critical transition is driven by a gradual shift in underlying disease parameters toward conditions favouring disease transmission, any outbreak is preceded both by an approach to criticality, which ends when transmission is exactly maintained by an effective reproduction number of one, and a delay as susceptible individuals accumulate in the population [10]. During the approach to criticality, the transmission system is expected to exhibit slower returns to mean (i.e. longer and longer chains of subcritical transmission) following the introduction of cases, which is a manifestation of critical slowing down [11]. The mathematical theory of critical slowing down has recently been worked out in detail for a number of specific disease systems [8–10,12]. General predictions of the theory are that cases will exhibit an increase in several summary statistics (table 1) as the critical point is approached [8,13,14]. These early warning signals would precede the increase in cases at the beginning of the supercritical period that triggers an alert in the moving epidemic method [7].

**Table 1.** Names and equations for 10 summary statistics used in these analyses. The correlation coefficient and *p*-value corresponding to testing between December 1981 and April 1993 is also given for each indicator.

statistic	formula	correlation coefficient (*τ*)	*p*-value
mean	$m_{i}(x_{j})$	1.000	0.157
variance	$m_{i}({\left(x_{j}-\text{mea}{\text{n}}_{j}\right)}^{2})$	0.791	0.039
(variance first difference)	$\text{varianc}{\text{e}}_{i}-\text{varianc}{\text{e}}_{i-1}$	0.461	0.153
autocovariance	$m_{i}(\left(x_{j}-\text{mea}{\text{n}}_{j})(x_{\;j-1}-\text{mea}{\text{n}}_{\;j-1}\right))$	0.890	0.008
(lag-1 autocorrelation)	$\frac{\text{autocovarianc}{\text{e}}_{i}}{{\left(\text{varianc}{\text{e}}_{i}\;*\;\text{varianc}{\text{e}}_{i-1}\right)}^{2}}$	0.739	0.064
(decay time)	$\frac{-1}{\text{log}(\text{min}(\text{max}(\text{autocorrelatio}{\text{n}}_{i},0),1))}$	0.739	0.033
(index of dispersion)	$\frac{\text{varianc}{\text{e}}_{i}}{\text{mea}{\text{n}}_{i}}$	0.709	0.063
(coefficient of variation)	$\frac{{\left({\text{variance}}_{i}\right)}_{0}^{.5}}{\text{mea}{\text{n}}_{i}}$	0.537	0.168
skewness	$\frac{m_{i}({\left(x_{j}-\text{mea}{\text{n}}_{j}\right)}^{3})}{{\left(\text{varianc}{\text{e}}_{i}\right)}^{1.5}}$	0.145	0.415
kurtosis	$\frac{m_{i}({\left(x_{j}-\text{mea}{\text{n}}_{j}\right)}^{4})}{{\left(\text{varianc}{\text{e}}_{i}\right)}^{2}}$	−0.017	0.505

This paper reports the first test of this theory on real rather than simulated data for a vector-borne disease. Here, we show the evidence of critical slowing down in a time series of monthly *Plasmodium falciparum* malaria case incidence leading up to a resurgence in Kericho, Kenya. The April 1993 resurgence event has been attributed to the parasite's development of resistance to chloroquine, the anti-malarial drug administered to workers as a control strategy on these plantations [15]. Thus, the proposed mechanism of the disease's return is a gradual underlying process––the increasing frequency of resistance in the population––rather than a sudden event like importation. In five of the 10 indicators, we detect increases that serve as early warning signals of malaria resurgence during the approach to criticality. Theory suggests this phenomenon may be a broadly applicable one.

## 2. Methods

**Figure 1.**
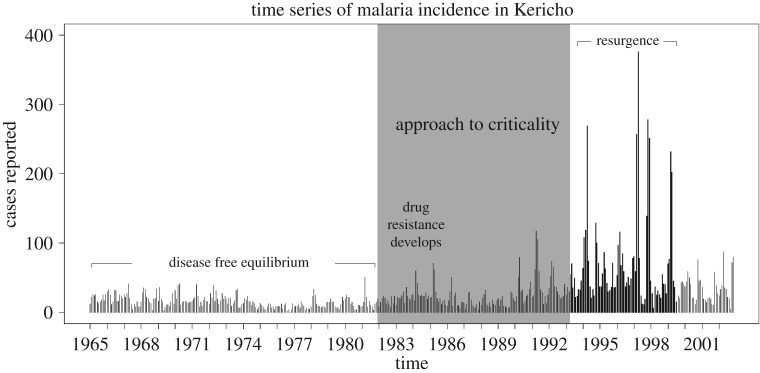
Monthly cases reported over the course of the time series. The approach to criticality preceding the 1993 outbreak is indicated by grey shading.

Data were obtained from two relatively isolated tea plantations for 1965 through 2002. The company-operated hospital provided case numbers based on stained blood smears from patients with suspected malaria [16,17]. Malaria reached epidemic levels in these areas during World War II, carried by soldiers returning from Ethiopia. In March 1948, a combination of proguanil prophylaxis and DDT house spraying brought malaria incidence down, and transmission was mostly controlled via the anti-malarial medication chloroquine until the April 1993 outbreak, when a spike in the number of cases exceeded all prior months by two and a half fold (figure 1) [15]. Operationally, we therefore define April 1993 as the boundary between subcritical and supercritical transmission. It is known that chloroquine remained fully effective against *P. Falciparum* through December 1981 [16]. Thus, we considered December 1981 to April 1993 to be the period during which the system approached criticality. We performed a sensitivity analysis to determine the effect of alternative endpoints for this window (electronic supplementary material, files S1 and S2). The theory of critical slowing down developed by O'Regan *et al*. [8] predicts that the indicators of critical slowing down should have increased during this time (table 1).

To test this prediction, from December 1981 to April 1993, we calculated each of these 10 leading indicators over a rolling window (i.e. the statistics were repeatedly calculated within a fixed size temporal subsample of the data, shifting the window by one month for each computation) (table 1). To better distinguish the signal of critical slowing down from background noise and periodic trends, we preprocessed the raw case data by detrending with a Gaussian kernel. The early warning signal itself was calculated so that for a moving window centred at time index *i,* a corresponding kernel weight *w_ij_* was assigned to the value of the statistic (mean, variance, etc.) at index *j* and then normalized so that $N_{i}=\sum_{j}w_{ij}$. Thus, for the time *i* and statistic *f*, the rolling window estimator *m*_i_ was defined as

$$m_{i}(f_{j}(x))=\sum_{\;j=i-b+1}^{i}\frac{w_{ij}f_{j}(x)}{N_{i}},$$

where the only tuning parameter is the bandwidth *b* = 40 (here set to 40 months, exceeding the period of malaria's seasonality to reduce any influence of seasonality on the overall trend). A sensitivity analysis shows that statistical results are relatively insensitive to the choice of bandwidth (electronic supplementary material, file S2). All analyses were conducted in R using the spaero package [18], a freely available package that provides a wide range of methods for investigating critical slowing down in time series. Data and code for reproducing analyses reported in this paper are available online at https://github.com/mjharris95/Kericho-EWS.git.

The performance of each indicator as an early warning signal was then assessed by performing significance tests on the rolling window statistics as follows. First, we determined the trend in each indicator over time by calculating Kendall's correlation coefficient, *τ*, between the calculated rolling window statistic at each time and the time index *i*. There are other statistics one could use (e.g. Spearman's correlation coefficient), but the use of Kendall's *τ* has become standard in the literature on anticipating critical transitions and our results are robust to choice of correlation coefficient (electronic supplementary material, file S3) [19–22]. The sign of *τ* indicates the direction of change, while the magnitude corresponds to the strength of the temporal trend. Unlike simulation studies, where an unlimited number of examples are available to investigate detectability, analysis based on data requires a means to assign statistical significance without access to replication. We used the following permutation approach to assess the significance of the estimated correlations. We first generated 10 000 permutations of case incidence during the approach to criticality (December 1981 to April 1993), thus preserving the general features of the original data (e.g. amplitudes of fluctuations) while eliminating underlying patterns of serial dependence. We then performed rolling window analyses on the permuted data to generate a null distribution of correlation coefficients for each indicator. The false positive rates of the 10 tests were estimated by finding the proportion of these permutations with a value for *τ* more extreme than that calculated for the Kericho data. The resulting *p*-values quantify the reliability of their corresponding statistics as early warning signals. In an applied surveillance setting, time series data would be updated regularly. To reflect this, we first included only data collected at least 96 months prior to the critical transition (April 1985) and repeatedly added an additional month of data, recalculating *p*-values for each month.

## 3. Results

**Figure 2.**
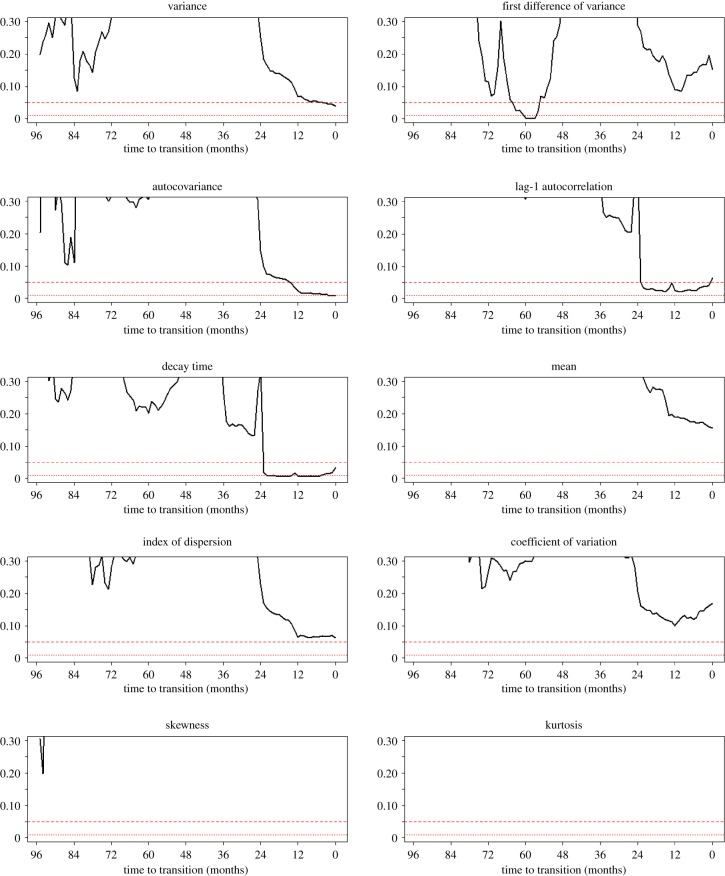
Time series. These plots give the *p*-value of the signal from each indicator, computed monthly starting in April 1985, 96 months prior to the notional month of critical transition, to April 1993. Red horizontal lines indicate *p*-values of 0.05 and 0.01 for reference.

The test values for all indicators except for kurtosis and skewness lie in the right tails of their null distributions, consistent with the prediction that critical slowing down results in an increase in these statistics (electronic supplementary material, file S4). Visually, it appears that most of the indicators exhibit intermittent periods of both upward and downward trend. The first difference of variance was the first indicator to return a low *p*-value (about 65 months prior to the critical transition), although the *p*-values for this indicator increased after a few months, remaining high for the remainder of the study (figure 2). The *p*-values for increases in decay time and lag-1 autocorrelation both dropped rapidly 24 months prior to the critical transition. The *p*-values for increases in autocovariance and variance also rapidly decreased 24 months prior to the critical transition and dropped below 0.05 fifteen and four months prior to the critical transition, respectively.

## 4. Discussion

These results show that simple summary statistics may serve as early warning signals of disease resurgence. Nine of the 10 statistical indicators increased during the approach to criticality, as predicted by the theory of critical slowing down, although only the increases in half of these indicators corresponded to low *p*-values (below 0.05) at any point in the 96 months preceding the critical transition. The first difference of variance was the first statistic to return early warning signals, followed by autocorrelation and decay time, then autocovariance, then variance. In the months immediately preceding the critical transition, the *p*-values for increases in the latter four declined sharply. This suggests that certain indicators may be more reliable early warning signals of critical slowing down at different times during the approach to criticality. The finding that variance and lag-1 autocorrelation are relatively robust early warning indicators is consistent with findings in simulation studies [8,12,19] and in other (non-disease) natural systems [11,20]. Our conclusion is that the statistical signatures of critical slowing down may be detected in ordinary surveillance data prior to disease resurgence and therefore should be incorporated into monitoring programmes and decision support for proactive response. The statistical features of the lag between the critical transition and disease resurgence, particularly in vector-borne disease systems, are not yet well understood [10].

There are qualifications to this conclusion. Our methods assume that the system is forced through a critical transition due to some slow-moving underlying mechanism, causing one or more disease transmission parameters to vary over time. The Kericho example meets these conditions, drawing from a nearly closed system without major human immigration, and undergoing a critical transition due to the gradual development of parasite resistance to chloroquine, with drug-resistant parasites becoming prevalent in the areas of Kenya starting in the early 1980s [23]. Vector-borne disease resurgence provoked by imported cases or a sudden change in the efficacy or administration of control measures is not expected to be detected in this manner. However, the accumulation of insecticide and pesticide resistance remains leading causes of malaria resurgence [24]. Moreover, in many areas, slow environmental forcing may drive additional changes in transmission, for instance as climate change shifts the geographic region suitable for malaria transmission [25]. Furthermore, this framework could be used to predict a critical transition toward elimination, provided that the system undergoes a gradual approach to criticality, for instance through increasing insecticide and bed net usage [8,9]. Thus, we think monitoring programmes based on critical slowing down might be used to inform intervention strategies by helping to distinguish between true changes in system stability and a temporary decrease in cases.

Although this study provides evidence that rolling window statistics may be used to anticipate disease resurgence, applications may be hampered by data limitations [12]. For instance, under-reporting, especially failure to detect asymptomatic carriers, may corrupt disease surveillance data. In our study, since the population eligible for healthcare remained constant over the course of the time series, inaccurate case reports are unlikely to vary in any systematic way, justifying our decision to use case reporting as a measure of incidence [16]. Moreover, theoretical studies suggest that early warning indicators based on critical slowing down are surprisingly robust to imperfect reporting [9,12,26]. Under intervention strategies that change case recovery rate, variance is expected to be robust to underreporting, whereas autocorrelation is expected to be sensitive to under-reporting [8]. Further theoretical studies are needed on the indicators of critical slowing down, particularly decay time, in the context of vector-borne disease systems. Investment in improved surveillance could alleviate the risk of costly resurgence responses. A related problem is that the models of the disease system approaching a critical transition used to identify potential early warning signals consider the total number of infectious individuals, whereas reported data (such as studied here) reflect the number of cases reported in a sampling interval (here, monthly hospital reports) [16,26]. In our case, since the resolution of the time series is a month, exceeding the initial infectious period of malaria, and the system slowly approaches resurgence, new cases are approximately equivalent to the total infectious individuals at a time point. But, such convenient alignment of case reporting and disease transmission may not be obtained in other situations. We recommend more extensive investigation of critical slowing down across infectious disease systems, particularly to identify which indicators most reliably predict critical slowing down and to analyse the effects of bandwidth and notional month of transition on early warning signal detection. Additional studies could also inform the selection of detection thresholds based on *p*-value and correction for testing based on multiple statistics that are not independent. Data requirements for such testing include regularly reported measures of incidence for tens to hundreds of infection generations, occurrence of outbreaks and a method for determining when the system reached the critical point. This last criterion could be satisfied either through parametric modelling of the data themselves or from independent information.

In conclusion, the results of this study support the use of rolling window calculations of indicators of critical slowing down in disease surveillance [8,9,12,14,26]. This study also demonstrates that early warning signals can be detected in monthly incidence data several years prior to a critical transition. The algorithmic nature of these methods points to the possibility that automated, model-independent disease forecasting systems could be developed for application directly to clinical data to assist in the detection of trends and predict disease resurgence, improving the efficiency of disease control campaigns and contributing to a welcome shift from reactive to preemptive response. For instance, we envision early warning systems that routinely monitor not only changes in the number of reported cases but also changes in these summary statistics and trigger an alert when a threshold is reached [8,9,12,14,26]. The detection of early warning signals could be followed by efforts to more specifically identify whether underlying features of the system are shifting (e.g. the development of drug resistance). In sum, being able to predict critical transitions might make malaria control (and other vertical eradication programmes) more efficient by prompting preemptive action prior to resurgence.

## Supplementary Material

Sensitivity Analysis of Testing Window Endpoints (Notional Month of Beginning of Approach to Criticality, Notional Month of Critical Transition)

## Supplementary Material

Sensitivity Analysis of Testing Parameters (Notional Month of Critical Transition, Bandwidth Size)

## Supplementary Material

Using Spearman's Correlation Coefficient (ρ)

## Supplementary Material

Indicators Over Time and Their Corresponding Null Distributions

## Data accessibility

Code used in the analyses and to generate figures and the corresponding data are available at https://github.com/mjharris95/Kericho-EWS.git
