# Supplementary material for: Early warning signals of malaria resurgence in Kericho, Kenya
Source: Biol Lett. 2020 Mar 18;16(3):20190713. doi: 10.1098/rsbl.2019.0713 (PMC7115183; doi:10.1098/rsbl.2019.0713)
Supplement: Sensitivity Analysis of Testing Window Endpoints (Notional Month of Beginning of Approach to Criticality, Notional Month of Critical Transition) [file rsbl20190713supp1.pdf]

Early Warning Signals of Malaria Resurgence in Kericho, Kenya

Mallory J. Harris, Simon I. Hay, and John M. Drake

Supplemental File 1: Sensitivity Analysis of Testing Window Endpoints (Notional Month of Beginning of Approach to Criticality, Notional Month of Critical Transition)

This supplement examines the sensitivity of the main result to two modeling choices: (1) the notional start of the approach to criticality, and (2) the notional start of the supercritical period (i.e., the first and final month of the time series used for early warning signal detection). We performed a panel analysis of the rolling window method for all combinations of approach to criticality start months from July 1981 through May 1982 and supercritical start months from November 1992 through September 1993. Correlation coefficients and p-values were calculated as in the main text.

The significance of the signal from variance and index of dispersion is greatest with an earlier start and end to the testing window.

The p-value for increases in autocovariance remains low across various testing windows with little variation.

The strength of the signal from lag-1 autocorrelation and decay time are greatest when the notional start of the supercritical period is earlier, suggesting that the critical transition occurs several months prior to the spike in cases.

The first difference of variance, mean, coefficient of variation, skewness, and kurtosis do not return a significant signal at the beginning of the supercritical period regardless of the endpoints of the testing window.

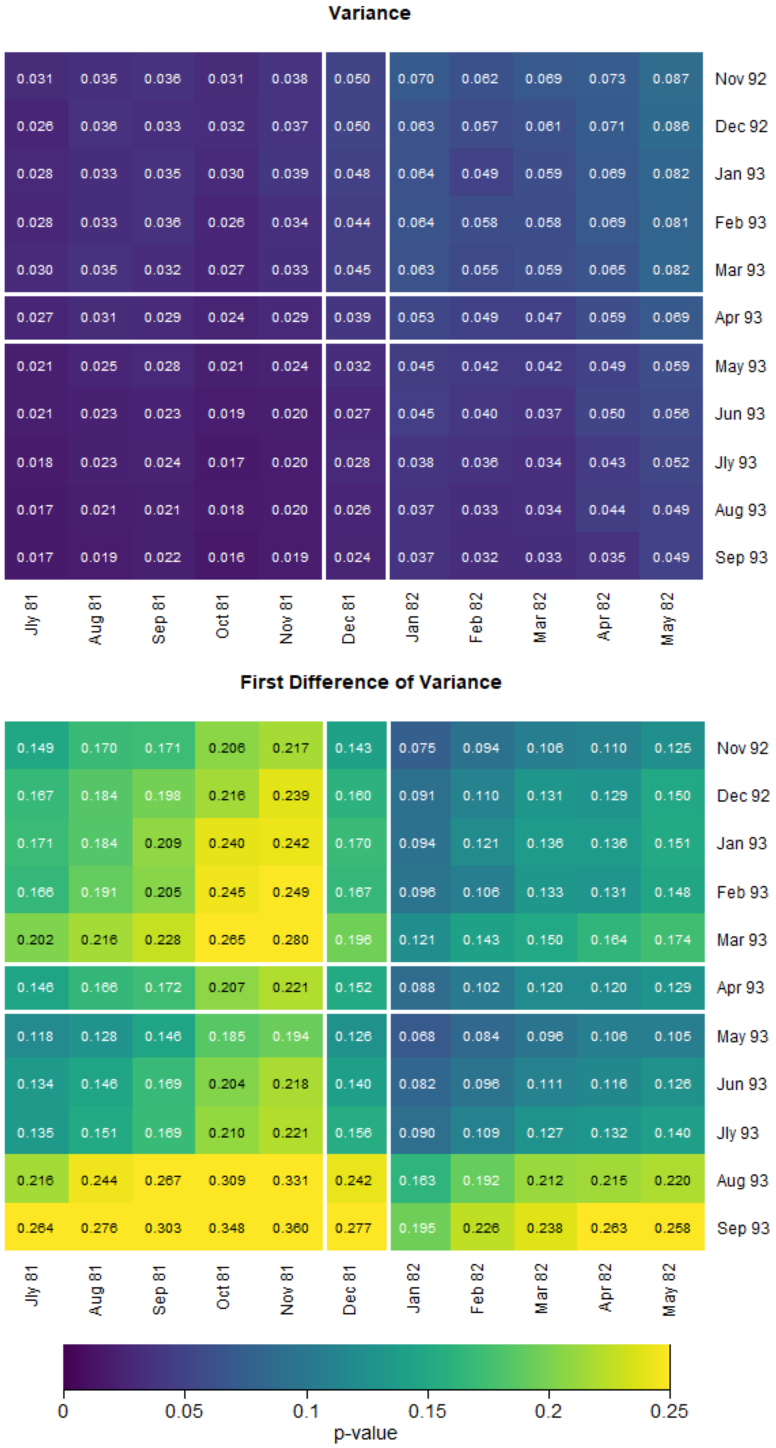

Supplemental Figures 1.1-1.2: The effect of the notional beginning of approach to criticality (x-axis) and notional month of critical transition (y-axis) on the resulting p-value for indicators of critical slowing down. Each cell displays the p-value for a given set of parameters and is shaded accordingly. The highlighted row/column identifies the values used in the main analysis (Dec 1981 – Apr 1993). The color key for p-values is displayed at the bottom of the plot and standardized across all indicators.

Autocovariance

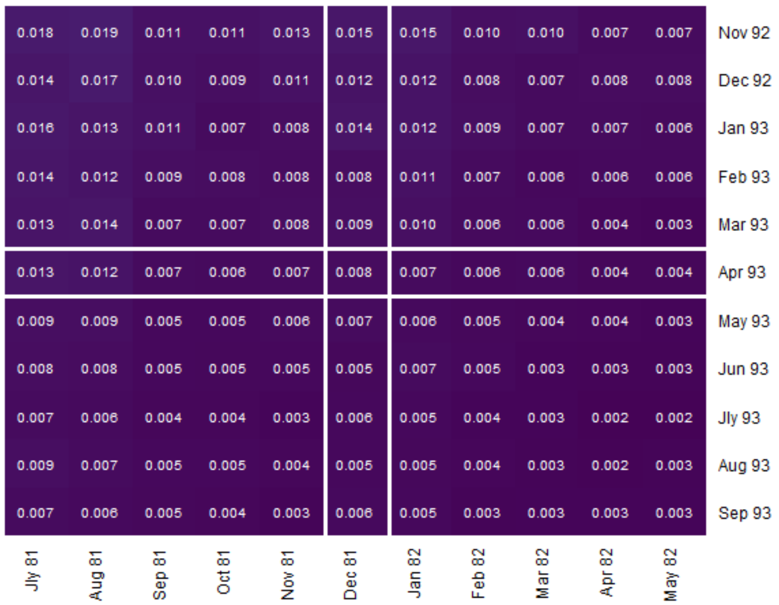

Lag-1 Autocorrelation

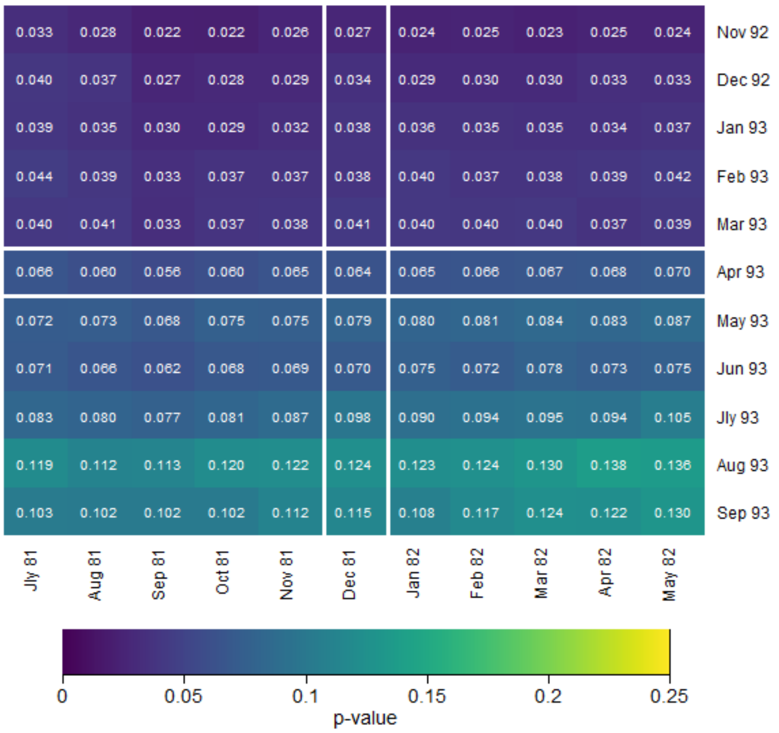

Supplemental Figures 1.3-1.4: The effect of the notional beginning of approach to criticality (x-axis) and notional month of critical transition (y-axis) on the resulting p-value for indicators of critical slowing down. Each cell displays the p-value for a given set of parameters and is shaded accordingly. The highlighted row/column identifies the values used in the main analysis (Dec 1981 – Apr 1993). The color key for p-values is displayed at the bottom of the plot and standardized across all indicators.

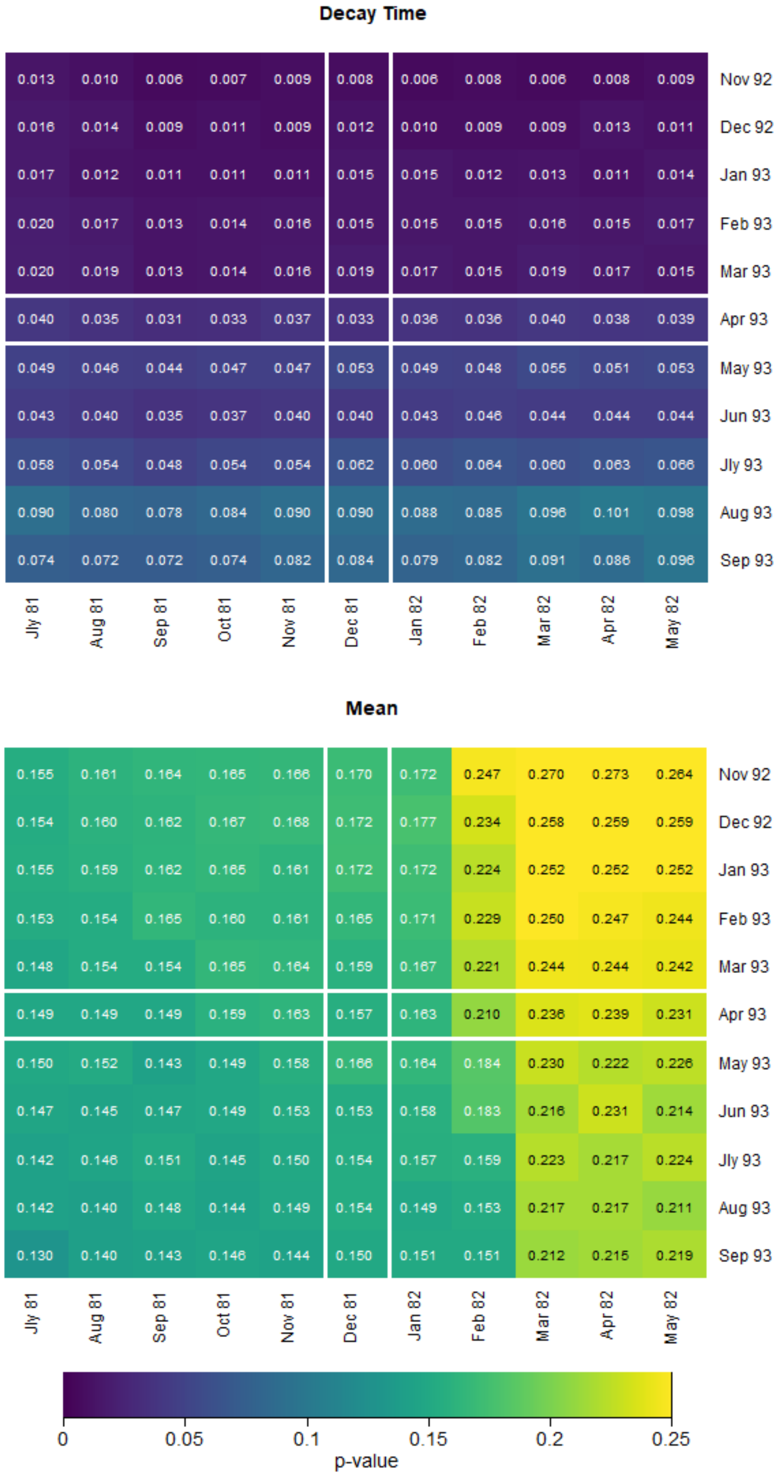

Supplemental Figures 1.5-1.6: The effect of the notional beginning of approach to criticality (x-axis) and notional month of critical transition (y-axis) on the resulting p-value for indicators of critical slowing down. Each cell displays the p-value for a given set of parameters and is shaded accordingly. The highlighted row/column identifies the values used in the main analysis (Dec 1981 – Apr 1993). The color key for p-values is displayed at the bottom of the plot and standardized across all indicators.

Early Warning Signals of Malaria Resurgence in Kericho, Kenya
Mallory J. Harris, Simon I. Hay, and John M. Drake
Biology Letters

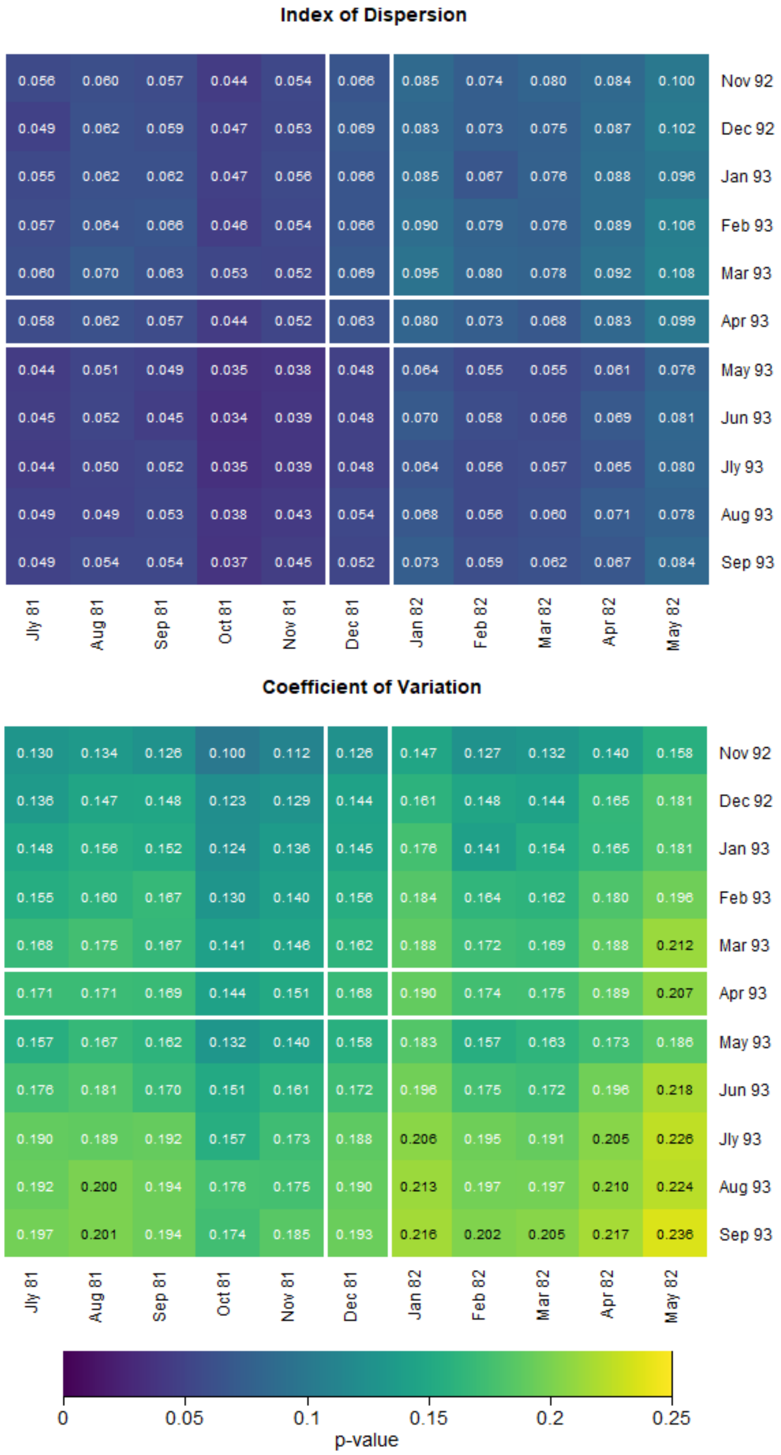

Supplemental Figures 1.7-1.8: The effect of the notional beginning of approach to criticality (x-axis) and notional month of critical transition (y-axis) on the resulting p-value for indicators of critical slowing down. Each cell displays the p-value for a given set of parameters and is shaded accordingly. The highlighted row/column identifies the values used in the main analysis (Dec 1981 – Apr 1993). The color key for p-values is displayed at the bottom of the plot and standardized across all indicators.

Skewness

	Jly 81	Aug 81	Sep 81	Oct 81	Nov 81	Dec 81	Jan 82	Feb 82	Mar 82	Apr 82	May 82
Nov 92	0.398	0.402	0.415	0.483	0.488	0.484	0.461	0.467	0.465	0.447	0.435
Dec 92	0.378	0.392	0.412	0.481	0.469	0.474	0.456	0.451	0.446	0.428	0.418
Jan 93	0.350	0.361	0.384	0.454	0.456	0.444	0.442	0.432	0.426	0.404	0.386
Feb 93	0.324	0.325	0.355	0.424	0.432	0.430	0.406	0.412	0.401	0.391	0.368
Mar 93	0.309	0.323	0.330	0.404	0.402	0.405	0.390	0.394	0.375	0.354	0.345
Apr 93	0.320	0.330	0.358	0.424	0.423	0.415	0.397	0.404	0.390	0.378	0.349
May 93	0.427	0.422	0.442	0.470	0.466	0.469	0.445	0.460	0.447	0.432	0.416
Jun 93	0.391	0.404	0.408	0.444	0.450	0.439	0.430	0.431	0.409	0.405	0.393
Jly 93	0.400	0.394	0.410	0.442	0.439	0.439	0.423	0.427	0.412	0.396	0.389
Aug 93	0.370	0.371	0.392	0.431	0.424	0.423	0.411	0.415	0.401	0.383	0.366
Sep 93	0.348	0.357	0.380	0.407	0.414	0.408	0.395	0.393	0.390	0.362	0.362

Kurtosis

	Jly 81	Aug 81	Sep 81	Oct 81	Nov 81	Dec 81	Jan 82	Feb 82	Mar 82	Apr 82	May 82
Nov 92	0.507	0.500	0.504	0.532	0.528	0.518	0.497	0.503	0.503	0.489	0.484
Dec 92	0.501	0.510	0.505	0.537	0.519	0.519	0.500	0.495	0.499	0.486	0.479
Jan 93	0.492	0.493	0.506	0.523	0.523	0.505	0.501	0.489	0.487	0.478	0.467
Feb 93	0.471	0.478	0.497	0.521	0.516	0.509	0.483	0.483	0.481	0.480	0.464
Mar 93	0.473	0.484	0.480	0.497	0.495	0.490	0.475	0.472	0.471	0.459	0.450
Apr 93	0.498	0.497	0.502	0.531	0.520	0.505	0.481	0.497	0.484	0.479	0.463
May 93	0.556	0.548	0.558	0.571	0.569	0.554	0.531	0.545	0.534	0.521	0.509
Jun 93	0.536	0.541	0.548	0.572	0.564	0.549	0.528	0.534	0.520	0.518	0.500
Jly 93	0.553	0.548	0.556	0.576	0.571	0.560	0.530	0.545	0.532	0.515	0.507
Aug 93	0.531	0.538	0.544	0.584	0.565	0.552	0.531	0.542	0.528	0.513	0.496
Sep 93	0.530	0.532	0.544	0.573	0.562	0.541	0.528	0.533	0.528	0.506	0.503

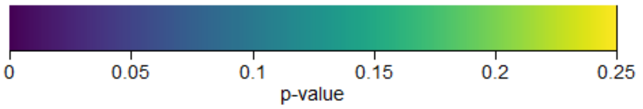

Supplemental Figures 1.9-1.10: The effect of the notional beginning of approach to criticality (x-axis) and notional month of critical transition (y-axis) on the resulting p-value for indicators of critical slowing down. Each cell displays the p-value for a given set of parameters and is shaded accordingly. The highlighted row/column identifies the values used in the main analysis (Dec 1981 – Apr 1993). The color key for p-values is displayed at the bottom of the plot and standardized across all indicator
