# Supplementary material for: Early warning signals of malaria resurgence in Kericho, Kenya
Source: Biol Lett. 2020 Mar 18;16(3):20190713. doi: 10.1098/rsbl.2019.0713 (PMC7115183; doi:10.1098/rsbl.2019.0713)
Supplement: Sensitivity Analysis of Testing Parameters (Notional Month of Critical Transition, Bandwidth Size) [file rsbl20190713supp2.pdf]

Early Warning Signals of Malaria Resurgence in Kericho, Kenya

Mallory J. Harris, Simon I. Hay, and John M. Drake

Supplemental File 2: Sensitivity Analysis of Testing Parameters (Notional Month of Critical Transition, Bandwidth Size)

This supplement examines the sensitivity of the main result to two modeling choices: (1) the selection of the bandwidth, and (2) the notional start of the supercritical period. We performed a panel analysis of the rolling window method for all combinations of supercritical start months from April 1992 through April 1994 and all bandwidths from b=35 to b=45. Correlation coefficients and p-values were calculated as in the main text.

Variance exhibits regular decreases in the significance of the signal with a larger bandwidth size and later notional month of critical transition. The p-value for increases in this indicator remains low across a range of bandwidth size and notional month of critical transition, indicating that it is relatively robust to variation in these testing parameters.

The first difference of variance exhibits the least regular trend in significance across testing parameter values, likely due to its large oscillations over time. For most values of the testing parameters, the second difference of variance does not return a significant signal.

Autocovariance is robust to parameter choice, with a low p-value across all testing parameters.

The significance of the signal from lag-1 autocorrelation increases with a later notional month of critical transition and greater bandwidth size. Lag-1 autocorrelation shows variation in the significance of its signal across testing parameters, although it returns predominantly low p-values across bandwidth size when the notional month of the critical transition is March 1993.

Decay time displays similar trends across parameter values to lag-1 autocorrelation, returning lower p-values than lag-1 autocorrelation at each testing parameter combination.

The significance of the signal from mean is greatest at intermediate bandwidth (between 38 and 41) and at later notional month of critical transition. This indicator does not return a significant signal at any combination of testing parameters.

The significance of the signal from index of dispersion is greatest at smaller bandwidth size and later notional month of critical transition.

The significance of the signal from coefficient of variation is greatest at smaller bandwidth size and extreme (i.e., nonintermediate) notional critical transition month. This indicator generally does not return significant values.

Skewness and kurtosis both do not return a significant signal, regardless of testing parameter values.

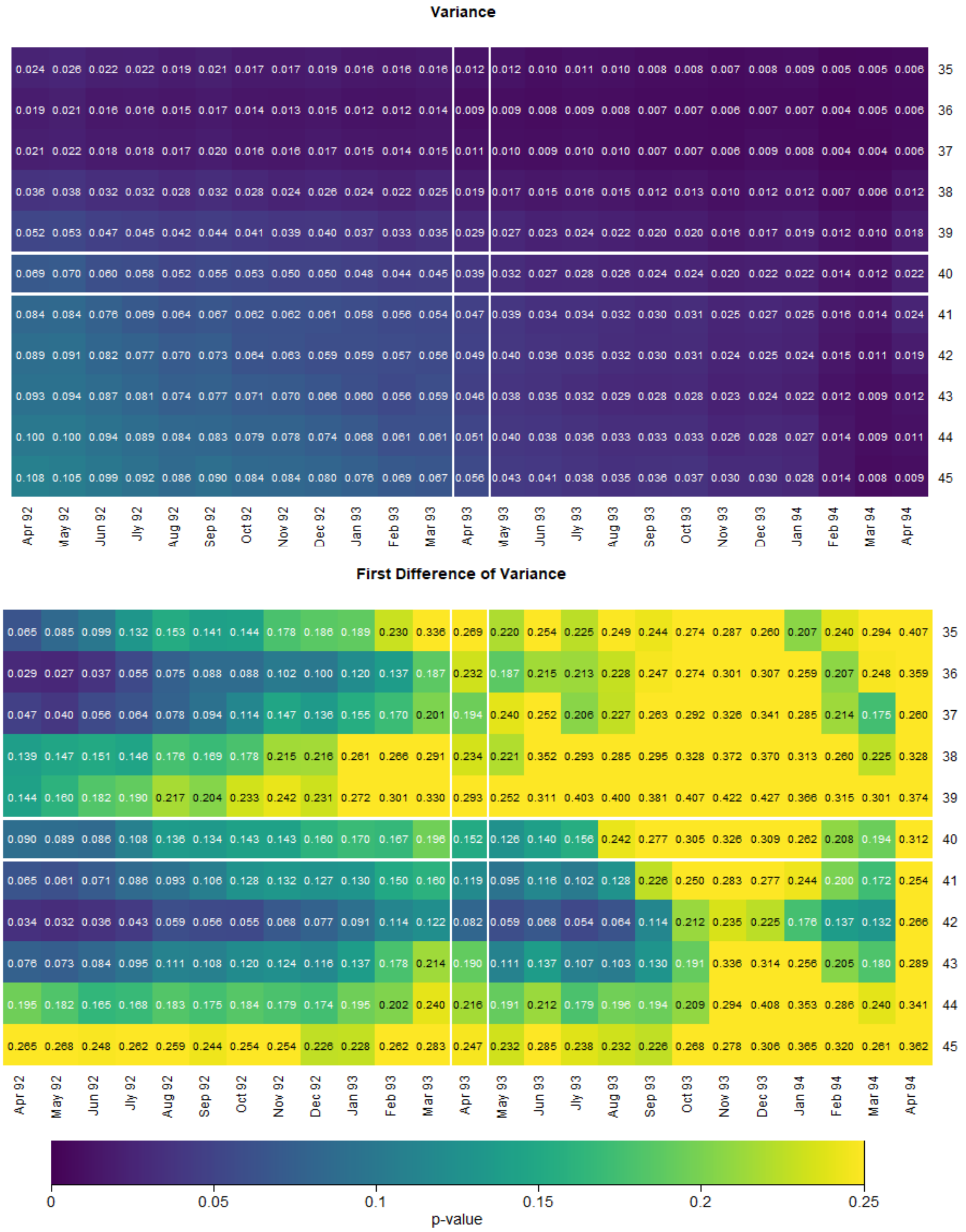

Supplemental Figures 2.1-2.2: The effect of notional start date (x-axis) and bandwidth (y-axis) on the resulting p-value for indicators of critical slowing down. Each cell displays the p-value for a given set of parameters and is shaded accordingly. The highlighted row/column identifies the values used in the main analysis (b=40 for the bandwidth and April 1993 for the start of the supercritical period). The color key for p-values is displayed at the bottom of the plot and standardized across all indicators.

Early Warning Signals of Malaria Resurgence in Kericho, Kenya
Mallory J. Harris, Simon I. Hay, and John M. Drake
Biology Letters

Autocovariance

	Apr 92	May 92	Jun 92	Jly 92	Aug 92	Sep 92	Oct 92	Nov 92	Dec 92	Jan 93	Feb 93	Mar 93	Apr 93	May 93	Jun 93	Jly 93	Aug 93	Sep 93	Oct 93	Nov 93	Dec 93	Jan 94	Feb 94	Mar 94	Apr 94
35	0.015	0.012	0.010	0.010	0.012	0.011	0.010	0.010	0.010	0.010	0.008	0.008	0.009	0.008	0.006	0.008	0.009	0.009	0.007	0.008	0.007	0.008	0.005	0.006	0.005
36	0.012	0.009	0.007	0.007	0.009	0.008	0.006	0.006	0.007	0.007	0.006	0.006	0.006	0.005	0.003	0.005	0.006	0.006	0.004	0.006	0.005	0.005	0.004	0.003	0.003
37	0.014	0.010	0.008	0.008	0.010	0.008	0.007	0.007	0.007	0.007	0.005	0.007	0.005	0.005	0.004	0.005	0.004	0.006	0.004	0.006	0.005	0.005	0.004	0.003	0.003
38	0.018	0.012	0.011	0.011	0.013	0.011	0.009	0.009	0.008	0.009	0.006	0.008	0.007	0.006	0.004	0.006	0.005	0.005	0.004	0.006	0.006	0.005	0.004	0.003	0.004
39	0.022	0.016	0.016	0.014	0.016	0.014	0.012	0.013	0.010	0.012	0.007	0.009	0.008	0.007	0.005	0.006	0.005	0.007	0.004	0.005	0.005	0.004	0.004	0.003	0.004
40	0.024	0.018	0.016	0.016	0.017	0.014	0.014	0.015	0.012	0.014	0.008	0.009	0.008	0.007	0.005	0.006	0.005	0.006	0.004	0.005	0.003	0.004	0.004	0.003	0.003
41	0.021	0.015	0.014	0.012	0.015	0.012	0.011	0.013	0.010	0.012	0.008	0.009	0.007	0.006	0.004	0.005	0.005	0.006	0.004	0.004	0.003	0.004	0.002	0.002	0.003
42	0.014	0.009	0.007	0.007	0.010	0.008	0.006	0.007	0.006	0.007	0.004	0.005	0.004	0.003	0.002	0.003	0.002	0.003	0.002	0.002	0.002	0.002	0.001	0.001	0.002
43	0.010	0.006	0.006	0.006	0.008	0.006	0.006	0.006	0.005	0.006	0.004	0.006	0.004	0.003	0.003	0.003	0.002	0.003	0.002	0.002	0.002	0.002	0.002	0.001	0.002
44	0.012	0.007	0.007	0.007	0.009	0.007	0.006	0.007	0.006	0.007	0.005	0.006	0.005	0.004	0.003	0.004	0.004	0.004	0.003	0.002	0.002	0.002	0.002	0.002	0.002
45	0.012	0.007	0.007	0.007	0.008	0.007	0.007	0.007	0.007	0.008	0.005	0.006	0.005	0.004	0.004	0.004	0.004	0.004	0.004	0.003	0.003	0.003	0.002	0.002	0.002

Lag-1 Autocorrelation

	Apr 92	May 92	Jun 92	Jly 92	Aug 92	Sep 92	Oct 92	Nov 92	Dec 92	Jan 93	Feb 93	Mar 93	Apr 93	May 93	Jun 93	Jly 93	Aug 93	Sep 93	Oct 93	Nov 93	Dec 93	Jan 94	Feb 94	Mar 94	Apr 94
35	0.038	0.030	0.028	0.030	0.033	0.037	0.032	0.029	0.048	0.059	0.055	0.052	0.070	0.070	0.062	0.078	0.087	0.081	0.080	0.071	0.081	0.099	0.131	0.094	0.266
36	0.042	0.031	0.029	0.030	0.032	0.036	0.032	0.028	0.051	0.063	0.061	0.061	0.075	0.078	0.070	0.086	0.093	0.082	0.081	0.075	0.090	0.110	0.143	0.105	0.283
37	0.036	0.030	0.029	0.029	0.030	0.031	0.031	0.028	0.045	0.055	0.055	0.057	0.073	0.081	0.069	0.090	0.096	0.090	0.088	0.079	0.093	0.116	0.150	0.112	0.302
38	0.031	0.026	0.025	0.025	0.026	0.027	0.028	0.027	0.038	0.047	0.048	0.050	0.070	0.079	0.071	0.093	0.102	0.096	0.094	0.090	0.098	0.123	0.154	0.120	0.325
39	0.028	0.023	0.022	0.024	0.024	0.026	0.025	0.026	0.036	0.042	0.045	0.046	0.065	0.080	0.069	0.094	0.112	0.101	0.103	0.097	0.108	0.131	0.166	0.129	0.343
40	0.026	0.022	0.021	0.024	0.026	0.026	0.024	0.027	0.034	0.038	0.038	0.041	0.064	0.079	0.070	0.098	0.124	0.115	0.115	0.108	0.119	0.143	0.185	0.142	0.361
41	0.024	0.020	0.019	0.023	0.025	0.026	0.022	0.024	0.031	0.038	0.039	0.040	0.061	0.075	0.068	0.097	0.128	0.122	0.126	0.121	0.131	0.153	0.203	0.152	0.376
42	0.022	0.020	0.018	0.024	0.024	0.026	0.022	0.023	0.028	0.035	0.037	0.042	0.053	0.070	0.062	0.095	0.130	0.119	0.131	0.127	0.140	0.164	0.221	0.165	0.393
43	0.025	0.023	0.022	0.026	0.028	0.029	0.025	0.028	0.030	0.038	0.039	0.045	0.062	0.074	0.068	0.094	0.127	0.123	0.128	0.133	0.147	0.176	0.233	0.174	0.406
44	0.024	0.021	0.019	0.023	0.025	0.028	0.023	0.026	0.030	0.033	0.034	0.040	0.055	0.072	0.066	0.091	0.114	0.110	0.117	0.116	0.148	0.183	0.250	0.181	0.416
45	0.021	0.020	0.018	0.022	0.024	0.026	0.022	0.027	0.031	0.034	0.034	0.036	0.052	0.067	0.063	0.086	0.105	0.102	0.109	0.113	0.131	0.180	0.266	0.181	0.432

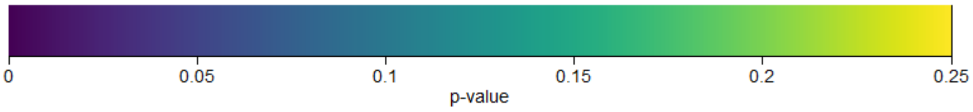

Supplemental Figures 2.3-2.4: The effect of notional start date (x-axis) and bandwidth (y-axis) on the resulting p-value for indicators of critical slowing down. Each cell displays the p-value for a given set of parameters and is shaded accordingly. The highlighted row/column identifies the values used in the main analysis (b=40 for the bandwidth and April 1993 for the start of the supercritical period). The color key for p-values is displayed at the bottom of the *plot and standardized across all indicators.*

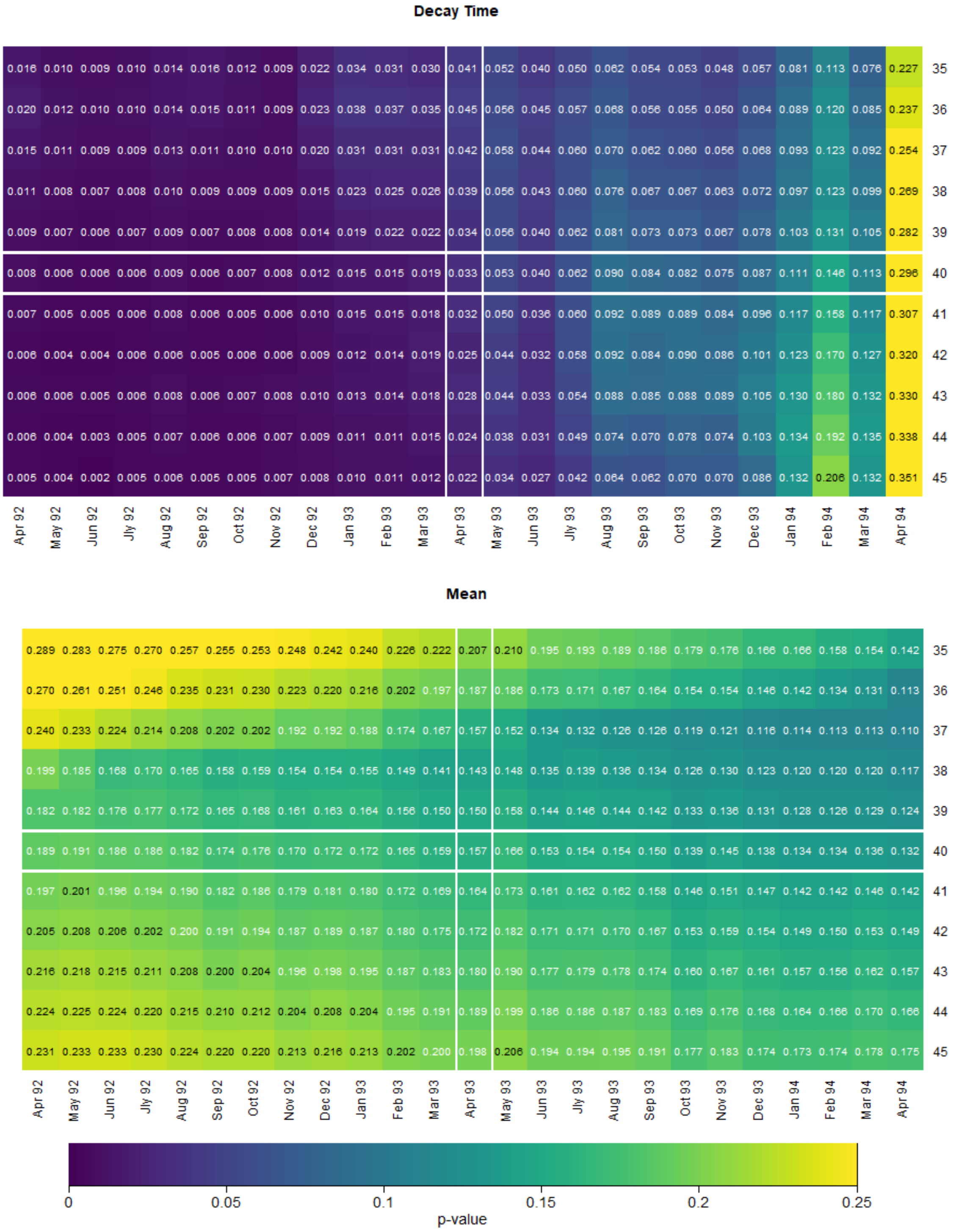

Supplemental Figures 2.5-2.6: The effect of notional start date (x-axis) and bandwidth (y-axis) on the resulting p-value for indicators of critical slowing down. Each cell displays the p-value for a given set of parameters and is shaded accordingly. The highlighted row/column identifies the values used in the main analysis (b=40 for the bandwidth and April 1993 for the start of the supercritical period). The color key for p-values is displayed at the bottom of the plot and standardized across all indicators.

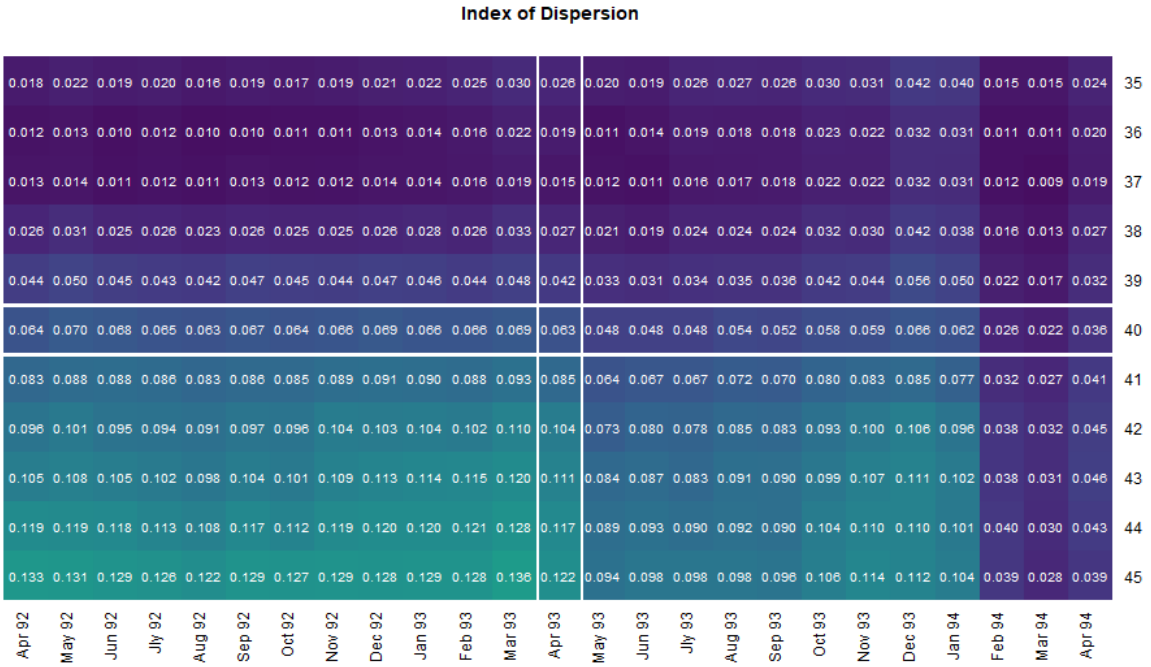

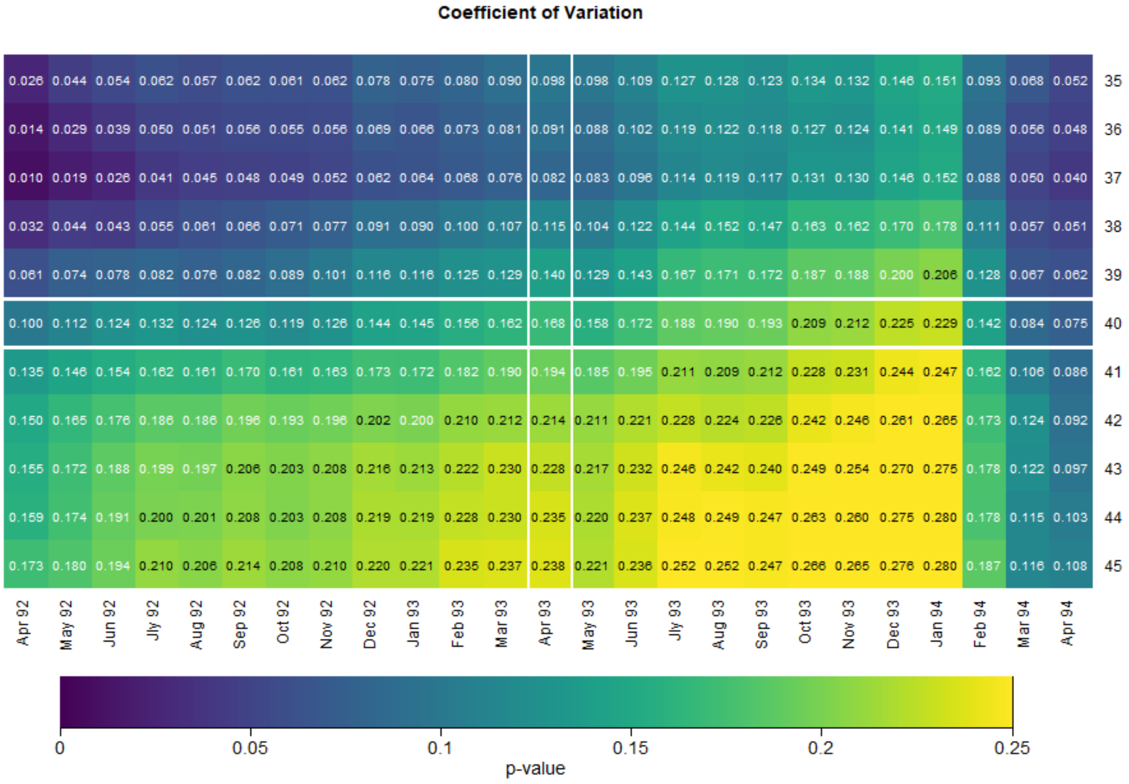

Supplemental Figures 2.7-2.8: The effect of notional start date (x-axis) and bandwidth (y-axis) on the resulting p-value for indicators of critical slowing down. Each cell displays the p-value for a given set of parameters and is shaded accordingly. The highlighted row/column identifies the values used in the main analysis (b=40 for the bandwidth and April 1993 for the start of the supercritical period). The color key for p-values is displayed at the bottom of the plot and standardized across all indicators.

Skewness

Apr 92	May 92	Jun 92	Jly 92	Aug 92	Sep 92	Oct 92	Nov 92	Dec 92	Jan 93	Feb 93	Mar 93	Apr 93	May 93	Jun 93	Jly 93	Aug 93	Sep 93	Oct 93	Nov 93	Dec 93	Jan 94	Feb 94	Mar 94	Apr 94	
0.561	0.559	0.552	0.559	0.543	0.548	0.534	0.525	0.525	0.504	0.494	0.452	0.460	0.512	0.479	0.474	0.463	0.455	0.413	0.391	0.382	0.420	0.472	0.472	0.350	35
0.557	0.557	0.548	0.554	0.539	0.547	0.531	0.521	0.521	0.495	0.490	0.456	0.461	0.510	0.476	0.468	0.457	0.446	0.409	0.392	0.386	0.408	0.452	0.454	0.346	36
0.544	0.545	0.535	0.537	0.525	0.531	0.515	0.503	0.501	0.478	0.472	0.441	0.452	0.496	0.459	0.455	0.443	0.431	0.400	0.384	0.383	0.399	0.431	0.441	0.333	37
0.535	0.533	0.523	0.529	0.518	0.525	0.507	0.498	0.491	0.468	0.461	0.429	0.445	0.492	0.456	0.452	0.438	0.424	0.393	0.377	0.372	0.392	0.416	0.428	0.324	38
0.528	0.524	0.514	0.520	0.508	0.515	0.497	0.487	0.477	0.451	0.444	0.417	0.429	0.480	0.446	0.442	0.426	0.412	0.384	0.369	0.366	0.377	0.400	0.415	0.323	39
0.523	0.519	0.511	0.515	0.501	0.507	0.489	0.484	0.474	0.444	0.430	0.405	0.415	0.469	0.439	0.439	0.423	0.408	0.376	0.362	0.359	0.373	0.386	0.402	0.318	40
0.509	0.506	0.497	0.500	0.484	0.492	0.472	0.464	0.456	0.426	0.412	0.379	0.396	0.447	0.416	0.421	0.406	0.389	0.360	0.346	0.344	0.360	0.360	0.384	0.305	41
0.501	0.499	0.488	0.495	0.478	0.486	0.464	0.452	0.445	0.415	0.396	0.366	0.378	0.441	0.401	0.407	0.394	0.381	0.352	0.335	0.335	0.350	0.347	0.372	0.297	42
0.485	0.484	0.472	0.478	0.462	0.472	0.447	0.431	0.426	0.398	0.385	0.358	0.369	0.427	0.389	0.392	0.376	0.367	0.342	0.327	0.330	0.341	0.334	0.359	0.289	43
0.470	0.465	0.453	0.457	0.439	0.450	0.428	0.411	0.411	0.385	0.376	0.352	0.362	0.418	0.380	0.383	0.368	0.357	0.336	0.318	0.317	0.334	0.322	0.341	0.278	44
0.452	0.451	0.441	0.445	0.425	0.434	0.410	0.395	0.397	0.368	0.361	0.333	0.352	0.406	0.371	0.373	0.360	0.348	0.324	0.305	0.306	0.325	0.309	0.326	0.278	45

Kurtosis

Apr 92	May 92	Jun 92	Jly 92	Aug 92	Sep 92	Oct 92	Nov 92	Dec 92	Jan 93	Feb 93	Mar 93	Apr 93	May 93	Jun 93	Jly 93	Aug 93	Sep 93	Oct 93	Nov 93	Dec 93	Jan 94	Feb 94	Mar 94	Apr 94	
0.652	0.639	0.637	0.636	0.617	0.624	0.611	0.604	0.607	0.591	0.594	0.562	0.575	0.631	0.620	0.628	0.620	0.616	0.605	0.596	0.605	0.626	0.699	0.742	0.372	35
0.633	0.626	0.621	0.621	0.604	0.612	0.593	0.588	0.587	0.574	0.579	0.555	0.569	0.616	0.609	0.615	0.607	0.602	0.590	0.582	0.593	0.610	0.684	0.726	0.357	36
0.614	0.605	0.598	0.600	0.588	0.595	0.578	0.571	0.569	0.554	0.560	0.539	0.554	0.599	0.590	0.598	0.590	0.582	0.568	0.563	0.575	0.588	0.667	0.704	0.339	37
0.590	0.581	0.576	0.578	0.566	0.574	0.555	0.551	0.552	0.538	0.540	0.520	0.532	0.583	0.574	0.581	0.573	0.564	0.549	0.544	0.554	0.567	0.649	0.686	0.326	38
0.573	0.565	0.559	0.558	0.548	0.556	0.535	0.531	0.533	0.522	0.524	0.504	0.519	0.570	0.564	0.570	0.562	0.550	0.538	0.536	0.542	0.549	0.628	0.669	0.309	39
0.555	0.546	0.542	0.543	0.534	0.540	0.519	0.518	0.519	0.505	0.509	0.490	0.505	0.554	0.549	0.560	0.552	0.541	0.529	0.528	0.536	0.540	0.609	0.653	0.301	40
0.542	0.534	0.529	0.531	0.522	0.528	0.510	0.506	0.508	0.494	0.495	0.480	0.492	0.540	0.532	0.540	0.535	0.532	0.516	0.516	0.526	0.527	0.594	0.638	0.293	41
0.535	0.527	0.523	0.527	0.516	0.526	0.508	0.506	0.510	0.493	0.494	0.479	0.493	0.536	0.527	0.535	0.533	0.528	0.516	0.514	0.523	0.522	0.587	0.629	0.291	42
0.529	0.518	0.516	0.520	0.508	0.519	0.502	0.502	0.504	0.489	0.493	0.475	0.491	0.534	0.526	0.533	0.531	0.527	0.517	0.512	0.522	0.522	0.579	0.617	0.283	43
0.516	0.506	0.504	0.508	0.494	0.506	0.488	0.490	0.495	0.481	0.488	0.476	0.486	0.522	0.513	0.522	0.517	0.514	0.505	0.501	0.510	0.512	0.563	0.592	0.270	44
0.504	0.495	0.494	0.497	0.483	0.496	0.477	0.476	0.482	0.470	0.477	0.464	0.476	0.511	0.505	0.510	0.506	0.501	0.491	0.489	0.501	0.501	0.551	0.574	0.259	45

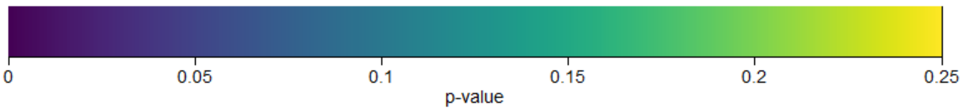

Supplemental Figures 2.9-2.10: The effect of notional start date (x-axis) and bandwidth (y-axis) on the resulting p-value for indicators of critical slowing down. Each cell displays the p-value for a given set of parameters and is shaded accordingly. The highlighted row/column identifies the values used in the main analysis (b=40 for the bandwidth and April 1993 for the start of the supercritical period). The color key for p-values is displayed at the bottom of the plot and standardized across all indicators.
