# Supplementary material for: Early warning signals of malaria resurgence in Kericho, Kenya
Source: Biol Lett. 2020 Mar 18;16(3):20190713. doi: 10.1098/rsbl.2019.0713 (PMC7115183; doi:10.1098/rsbl.2019.0713)
Supplement: Using Spearman's Correlation Coefficient (ρ) [file rsbl20190713supp3.pdf]

Early Warning Signals of Malaria Resurgence in Kericho, Kenya

Mallory J. Harris, Simon I. Hay, and John M. Drake

Supplemental File 3: Using Spearman's Correlation Coefficient (ρ)

This supplement examines the sensitivity of the main result to the correlation coefficient used to the quantify the increase in each indicator over time. Kendall's τ is generally used in early warning signal detection and therefore used in the main text. Pearson correlation coefficient (r) is unsuitable for these analyses, as it quantifies the strength of linear relationships instead of monotonic relationships (compared to Kendall's τ and Spearman's ρ). By repeating the analyses using Spearman's correlation coefficient (ρ), we found that analyses based on Spearman's ρ generally returned slightly less significant signals, although our results are robust to choice of correlation coefficient (Supplemental Figure 3). The data for these analyses are available at https://github.com/mjharris95/Kericho-EWS.

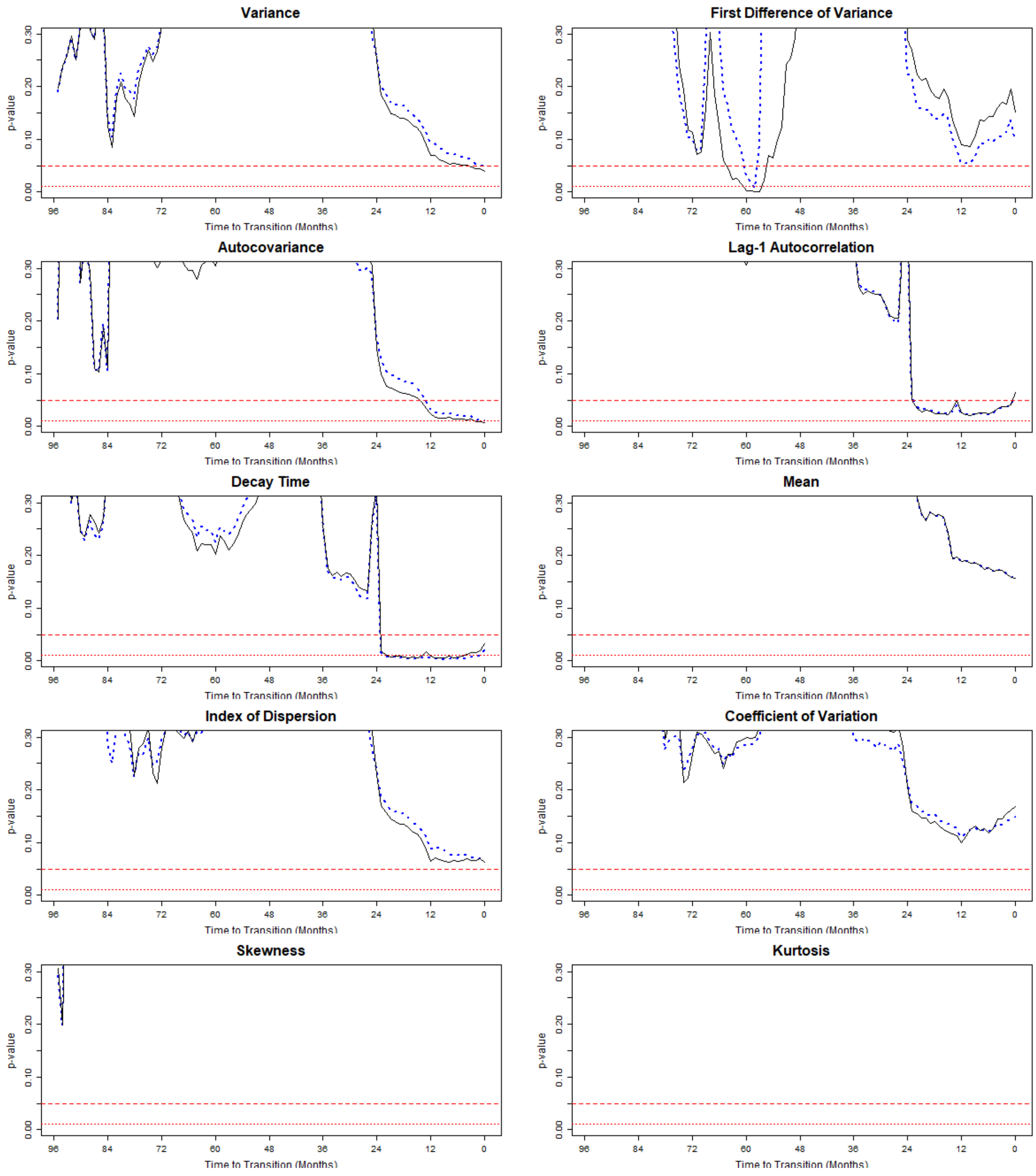

Supplemental Figure 3: Analogous to figure 3, these plots give the p-value of each signal, computed monthly starting in April 1985, 96 months prior to the notional month of critical transition, April 1993. The red horizontal lines indicate p-values of .05 and .01 for reference. The solid black lines give the signal based on Kendall's τ and the dashed blue lines give the signal based on Spearman's ρ
