# Supplementary material for: Early warning signals of malaria resurgence in Kericho, Kenya
Source: Biol Lett. 2020 Mar 18;16(3):20190713. doi: 10.1098/rsbl.2019.0713 (PMC7115183; doi:10.1098/rsbl.2019.0713)
Supplement: Indicators Over Time and Their Corresponding Null Distributions [file rsbl20190713supp4.pdf]

Early Warning Signals of Malaria Resurgence in Kericho, Kenya

Mallory J. Harris, Simon I. Hay, and John M. Drake

Biology Letters

Supplemental File 4: Indicators Over Time and Their Corresponding Null Distributions

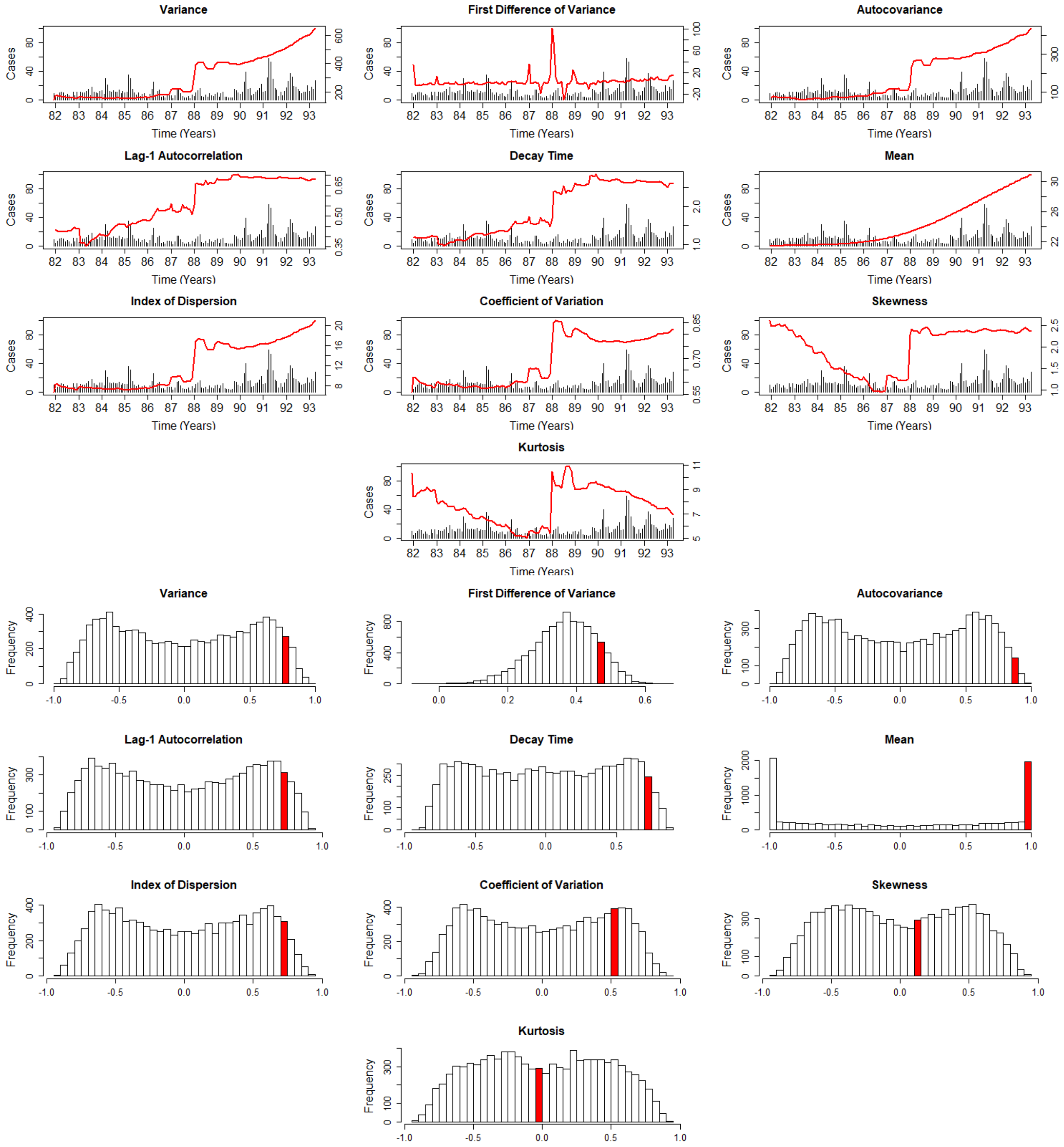

Supplemental Figure 4: Trajectories of the rolling window statistics over time (red line) plotted over monthly case incidence (barplot). [Bottom]: The null distribution of each indicator plotted as a histogram with the bin containing the test statistic (i.e. correlation coefficient Kendall's τ calculated for December 1981 - April 1993) is indicated in red. The significance of each indicator's correlation coefficient value for the Kericho data is measured as the proportion of total area to the right of each test value in its corresponding null distribution.
